# Supplementary material for: Learning curve and surgical outcome of robotic assisted colorectal surgery with ERAS program
Source: Sci Rep. 2022 Nov 29;12:20566. doi: 10.1038/s41598-022-24665-w (PMC9709162; doi:10.1038/s41598-022-24665-w)
Supplement: Supplementary file 1 — Supplementary Information 1. [file 41598_2022_24665_MOESM1_ESM.zip › LSC Raw data-Ñ[▒K/100/H_ñw╝╨░O▒Kñσ.pdf]

正式摘要(電子簽章：0000002022251525)

W52 / W52 ☐英文標題(注意:無英文姓名-請補病人基本檔!) ☐【讀取】憑證

臺中榮民總醫院

出院病歷摘要

轉入號 : 轉出號 :

[redacted]

**(8)**流水編號	
**(9)**入院日期	109/10/28 (2020) 直腸外科 W52 - 091
**(10)**轉科日期	
**(11)**出院日期	109/11/02 (2020) 住院天數計 5 日

診斷

- 入院 :1. Sigmoidal cancer
  2. Type II DM, under OHA
- 出院 :1. Sigmoidal cancer, s/p laparoscopic Anterior resection and anastomosis on 10/29
  2. Type II DM, under OHA

主訴

- Bloody stool was noted while Augest,2020

病史

- This 58 y/o female has past history of type II DM.
  This time, she got some bloody stool in Augest. Then, she had been to 員基 hospital for help, where colonscopy was performed and sigmoidal cancer was suspected. There was no fever, headache, dyspnea, abdominal discomfort nor urine frequency change. Abdominal CT showed sigmoidal Ca. with regional metastatic lymphadenopathy. Imaging stage T1-2N1M0. Under the impression of sigmoidal cancer, she was admitted to our ward for surgery and further management.

體檢發現

- CEA 1.3
  - General appearance :
    - easy looking , conscious clear , without cardiopulmonary distress
    - Vital signs自動帶入date 體溫(BT) 脈搏(P) 呼吸(R) 血壓(BP) 身高(BH) 體重(BW) BMI
      20201028 36.4℃ 75次/分 18次/分 134/80mmHg 163cm 64kg 24.1
    - Vital signs: B.P. 134/80 mmHg , P.R. 75 /min , R.R. 18 /min , B.T. 36.4 ℃
  - HEENT :
    - Eyes: no anemic conjunctiva, no icteric sclera, normal EOM
    - ENT: no otorrhea, no rhinorrhea, no erythematous mucosa, no injected tonsil, no deformity
  - Neck: supple, freely movable, trachea midline,no deformity, no engorged jugular vein , no palpable thyroid or lymph node enlargement
  - Chest:
    - Inspection: Grossly normal, symmetric and fully expansion, no accessory muscle used, no operation scar, no spider angiomata, no gynecomastia
    - Palpation: no palpable mass or nodule, no subcutaneous emphysema, no tenderness
    - Ausculation: clear breathing sound
  - Heart:
    - Palpation: no thrill, no heave
    - Percussion: normal heart size
    - Ausculation: regular heart beat, normal S1, S2, no gallop, no murmur
  - Extremities: No deformity, full ranged of motion without limitation, no clubbing finger or nail cyanosis, no pitting edema, no ulcer or pigmentation, no obvious skin lesion, no varicose vein, no bone, joint or muscle tenderness

- **CRS CONDITION**
  - Karnofsky performance status: (100) Normal, no complaints, no evidence of disease
  - PE of Abdomen:
  - CEA: 1.32 ng/ml
  - Imaging Studies:
  - Colonoscope report: Advanced colorectal cancer; Sigmoid colon ; ulcerative type, 35cm FAV
  - Figures:
    1. colon & rectum

手術日期及方法

- 手術日期及方法20201029 Laparoscopic Anterior resection and anastomosis - malignant

住院治療經過

- After admission, operation was done on 10/29. Try clear liquid diet on 10/29 for ERAS case. Try semiliquid diet on 10/31. Due to relative stable condition, MBD was permitted and OPD f/u is scheduled for further treatment.

合併症

一般檢查

特殊檢查

病理報告

其它

出院時情況

- 3.改門診治療

出院指示

藥名	劑量	劑量單位	用法	頻次	數量	數量單位	現況	天數	單位
Pantoloc tab 40mg	1	TAB	PO	QDAC	8	TAB	TH	8	天
Ultracet tab	0.5	TAB	PO	Q6H	16	TAB	TH	8	天

出院計劃

主治醫師 2062F 蔣鋒帆 / 住院醫師 2310K 陳瑋夫

正式摘要(電子簽章：000000202251525)

W52 / W52

doctorRank:B
